# Glycolipid Transfer Protein Expression Is Affected by Glycosphingolipid Synthesis

**DOI:** 10.1371/journal.pone.0070283

**Published:** 2013-07-24

**Authors:** Matti A. Kjellberg, Peter Mattjus

**Affiliations:** Biochemistry, Department of Biosciences, Åbo Akademi University, Turku, Finland; University of Geneva, Switzerland

## Abstract

Members of the glycolipid transfer protein superfamily (GLTP) are found from animals and fungi to plants and red micro-alga. Eukaryotes that encode the glucosylceramide synthase responsible for the synthesis of glucosylceramide, the precursor for most glycosphingolipids, also produce GLTPs. Cells that does not synthesize glucosylceramide neither express GLTPs. Based on this genetic relationship there must be a strong correlation between the synthesis of glucosylceramide and GLTPs. To regulate the levels of glycolipids we have used inhibitors of intracellular trafficking, glycosphingolipid synthesis and degradation, and small interfering RNA to down-regulate the activity of glucosylceramide synthase activity. We found that GLTP expression, both at the mRNA and protein levels, is elevated in cells that accumulate glucosylceramide. Monensin and brefeldin A block intracellular vesicular transport mechanisms. Brefeldin A treatment leads to accumulation of newly synthesized glucosylceramide, galactosylceramide and lactosylceramide in a fused endoplasmic reticulum-Golgi complex. On the other hand, inhibiting glycosphingolipid degradation with conduritol-B-epoxide, that generates glucosylceramide accumulation in the lysosomes, did not affect the levels of GLTP. However, glycosphingolipid synthesis inhibitors like PDMP, NB-DNJ and myriocin, all decreased glucosylceramide and GLTP below normal levels. We also found that an 80% loss of glucosylceramide due to glucosylceramide synthase knockdown resulted in a significant reduction in the expression of GLTP. We show here that interfering with membrane trafficking events and simple neutral glycosphingolipid synthesis will affect the expression of GLTP. We postulate that a change in the glucosylceramide balance causes a response in the GLTP expression, and put forward that GLTP might play a role in lipid directing and sensing of glucosylceramide at the ER-Golgi interface.

## Introduction

Glycosphingolipids (GSLs) are lipids with a hydrophobic ceramide backbone and varying hydrophilic carbohydrate moieties as a headgroup. The majority of the GSLs localize to the extracellular leaflet of the plasma membrane, where they take part in diverse cellular processes, such as cell adhesion, signaling and sorting events [1]. The glycosylation of complex GSLs takes place on the luminal side of the Golgi apparatus [2]–[5]. The precursor of most of the complex GSLs, glucosylceramide (GlcCer), is produced on the cytosolic side of the cis-Golgi membranes from ceramide and an activated UDP-glucose, by the UDP-glucose:ceramide glucosyltransferase, glucosylceramide synthase (EC 2.4.1.80, GlcCerS) [6], [7]. Ceramide can also be galactosylated by the 2-hydroxyacylsphingosine 1-beta-galactosyltransferase (EC 2.4.1.45, GalCerS) on the luminal membrane surface of the endoplasmatic reticulum (ER), to produce galactosylceramide (GalCer) [8], [9]. Lactosylceramide is produced from GlcCer in the lumen of the Golgi by glucosylceramide β1→4 galactosyltransferase (EC 2.4.1.274, LacCerS) [10]. LacCerS appears to be present both in the Golgi cisternae and in the trans-Golgi network [11]–[13]. Sphingomyelin (SM) is also synthesized *de novo* from ceramide, but this occurs in the lumen of the trans-Golgi compartment as well as on the plasma membrane. It is unclear which of these pathways this is responsible for the *de novo* synthesis of SM [5].

The glycolipid transfer protein (GLTP) is a cytosolic protein [14] that catalyzes the transport of both sphingoid and glycerol based glycolipids *in vitro* [15], [16]. GLTP does not transfer phospholipids, SM or neutral lipids [17], [18]. GLTP with its all-alpha helical fold and novel structural motif, is the founding member for a new protein superfamily in eukaryotes [19]. GLTP is found from animals and fungi to plants and red micro alga. Several homologues to mammalian GLTP have been found in various species [20]–[23], including the human FAPP2 (phosphoinositol 4-phosphate adaptor protein 2), that contains a GLTP-motif, and has been shown to mediate the transfer of GlcCer from early Golgi to distal Golgi compartments [23], [24].

To this date, it is not known how GLTP is regulated in the cell, and if GLTP is involved in intracellular glycolipid synthesis and trafficking. Based on a point mutational data study, GLTP has been indirectly suggested to be involved in the intracellular sensoring and/or trafficking of GlcCer [25]. Further support for this suggestion comes, not only from the seemingly strong evolutionary connection between GlcCer and GLTP as mentioned above, but also from the fact that GlcCer is synthesized on the outer side of the Golgi membrane, leaving the lipid accessible for interaction with the cytosolic GLTP. Previously, we have shown that overexpression of GLTP in HeLa cells, and in this study human skin fibroblast cells (HSFs), leads to increased GlcCer levels while siRNA-mediated GLTP depletion does not alter GlcCer levels significantly [14]. This indicates that GLTP does not like FAPP2 have a direct role in the synthesis of higher glycosphingolipids, because depletion of GLTP does not affect their synthesis [14]. We have also demonstrated *in vitro* that GLTP interacts with the vesicle-associated protein-associated protein VAP-A, an ER protein that interacts with the FFAT-like motif (two phenylalanines in an acidic tract) in GLTP and other cytosolic lipid-binding proteins [26]. In a recent screening study, weaker FFAT-like VAP association motifs were found, both in FAPP2 as well as in different GLTPs that might suggest a possibility for different strengths of ER targeting [27].

The investigation of intracellular lipid transfer processes is challenging. However, with the use of different inhibitors of glycolipid homeostasis and intracellular trafficking, we have now been able to study the connection between GLTP and changes in the cellular amounts of GSLs. In this study we have also used RNA interference to down-regulate the expression of GlcCerS and subsequently cause inhibition of GlcCer synthesis. We found that similar to the GlcCer synthesis inhibitors the amount of GLTP dropped significantly when less GlcCer was synthesized. It should be noted that the synthesis of ceramide in mammals is carried out by six distinct ceramide synthases each responsible for the production of ceramides with different chain lengths, making their down regulation complex [28], [29].

For the first time, we show that brefeldin A (BFA) and monensin treatment of HSFs results in significantly higher GLTP levels. Monensin and BFA both inhibit vesicular transport and lead to an increased synthesis of simple GSLs [30], [31]. To inhibit the retrograde GSL pathway we used the drug conduritol-B-epoxide (CBE). CBE treatment results in an accumulation of GlcCer in the lysosomes [32]. However, we did not observe any increase in the GLTP level in cells treated with CBE. Treatment of HSFs with myriocin, an inhibitor of serine palmitoyltransferase, the first step in sphingosine biosynthesis [33], decreased both GlcCer and GLTP levels below control values. The decrease in GLTP was also seen in cells treated with the GlcCer synthesis inhibitors PDMP and NB-DNJ. We also found that a loss of GlcCer caused by GlcCerS down-regulation significantly reduced the expression of GLTP. BFA and monensin treated GlcCerS knockdown HSF cells that consequently accumulated less GlcCer in the fused ER-Golgi stacks also had a lower expression of GLTP. We show here that interfering with membrane trafficking events and simple neutral glycosphingolipid synthesis will greatly affect the responses in GLTP. Taken together, based on these results, we suggest a role sensory role for GLTP close to or at the site of GlcCer synthesis.

## Materials and Methods

### Materials

The polyclonal antibody against human GLTP has previously been described [14]. The rabbit anti-beta-actin antibody was from Rockland Immunochemicals (Gilbertsville, PA, USA) and the secondary peroxidase conjugated rabbit anti-goat antibody was from Thermo Scientific (Waltham, MA, USA). Bovine brain sphingomyelin, Gaucher liver GlcCer, bovine butter lactosylceramide (LacCer), and bovine brain total galactosylcerebrosides (containing both hydroxylated GalCer and non-hydroxylated GalCer) were purchased from Avanti Polar Lipids (Alabaster, AL, USA). Methanol, chloroform, hexane, acetone, acetic acid and 2-propanol were from Avantor Performance Materials (formerly J.T. Baker) (Center Valley, PA USA). Monensin, brefeldin A (BFA) and N-butyldeoxynojirimycin (NB-DNJ) were obtained from Toronto Research Chemicals (North York, ON, Canada). Myriocin, 1-Phenyl–2-decanoylamino-3-morpholino-1-propanol (DL-*threo* PDMP) and protease inhibitor cocktail were purchased from Sigma (St. Louis, MO, USA). ^3^H-sphinganine was a kind gift from Dr. Tony Futerman, Weizmann Institute of Science, Israel. ^3^H-palmitic acid was a kind gift from Dr. J. Peter Slotte, Åbo Akademi University, Finland. Conduritol-B-epoxide (CBE) was purchased from Merck Chemicals (Calbiochem) (Darmstadt, Germany) and tunicamycin was purchased from Enzo Life Sciences (Farmingdale, NY, USA). Stealth RNAi and a Stealth RNAi negative control were obtained from Invitrogen (Carlsbad, CA, USA) and used for the transient down-regulation of human GlcCerS (NM_003358).

### Cell Culture

Human skin fibroblasts (HSF, GM08333) were obtained from Coriell Institute for Medical Research (Camden, NJ, USA). Cells were grown and treated in Dulbecco’s modified Eagle’s medium, (DMEM, Sigma-Aldrich, St. Louis, MO, USA) supplemented with penicillin/streptomycin, 2 mM L-glutamine and 10% fetal calf serum and grown at 5% CO_2_, 37°C.

### Stealth RNAi and Knockdown Experiments

HSF cells were grown to 50% confluence in 35 mm dishes and transiently transfected with 75 pmol of GlcCerS siRNAs with Lipofectamine 2000 (Invitrogen) according to the manufacturer’s instructions. The specific siRNA primers were, sense strand, CAGGUGUCUCUCUUCUGAAACCACU and anti-sense, AGUGGUUUCAGAAGAGAGACACCUG. The cells were cultured for 48 h, where after they were analyzed or treated accordingly (see results).

### Western Blotting

Cells were grown until 80% confluency before each experiment. Cells selected for Western blotting analysis were washed twice in phosphate buffered saline (PBS), pH 7.4, after treatment completion and suspended in a lysis buffer (50 mM NaH_2_PO_4_, 300 mM NaCl, 10 mM imidazole, 0.05% Tween-20, 0.5 mM PMSF, 1× protease inhibitor cocktail, 1 mM dithiotreithol, pH 8.0). Cells were sonicated on ice 6 times for 10 seconds using a Branson 250 probe sonifier (Emerson Industrial Automation, St. Louis, MO, USA). The protein concentration of the whole cell lysates was determined using the Lowry method [34]. For the detection of GLTP and loading controls, an amount corresponding to 50 µg of total protein was analyzed using standard Western blotting procedures, using the anti-GLTP and anti-beta-actin antibodies described above. The Western blotting results shown here are one representative blot from at least three independent experiments with similar results.

### ^3^H-sphinganine and ^3^H-palmitic Acid Labeling of Cells, Subsequent Treatments and Lipid Extraction

HSF cells were cultured to 80% confluency and labeled with 0.33 µCi/ml of ^3^H-sphinganine or 1.00 µCi/ml ^3^H-palmitic acid (dissolved in ethanol and added to the culture media) for 15 minutes at 37°C, after which they were treated as described below. BFA/monensin treatment was done for varying times and concentrations (see results). In the sphingolipid synthesis inhibitor co-treatment experiments, cells were pre-incubated with either NB-DNJ (250 µM), PDMP (50 µM) or myriocin (25 µM) for 2 hours before addition of ^3^H-sphinganine (to NB-DNJ and PDMP treated cells) or ^3^H-palmitic acid (to myriocin treated cells) and BFA (0.01 µg/ml) or monensin (5 µg/ml). In the CBE treatment experiments, HSF cells were treated with CBE (250 µM) and labeled with ^3^H-sphinganine for 5 days, and relabeled with radiolabeled precursor every 24 hours. To be able to follow the effects of the myriocin inhibition of sphingolipid synthesis the radionuclide has to be in palmitic acid. Myriocin inhibits the serine palmitoyltransferase, the reaction between palmitoyl-CoA and L-serine, one step before the ceramide synthase catalyzed reaction between sphinganine and fatty-acyl CoA yielding dihydroceramide. The ^3^H-sphingasine-label would not report the effects of myriocin.

After treatment, cells were washed three times in PBS, pH 7.4. Lipids were extracted directly from the culture dishes using hexane:2-propanol (3∶2, v/v) and the extracted lipids were dried under a nitrogen stream. Lipids were re-dissolved in hexane:2-propanol and analyzed on high performance thin-layer chromatography (HPTLC) silica plates (Whatman, UK) using a developing solvent containing chloroform:methanol:acetone:acetic acid:water (10∶2:4∶2:1). Identification of the different lipid species was done using lipid standards run in parallel with the samples. Visualization of the lipid migration was done using an iodine chamber, or by spraying with orcinol (0.2% orcinol in a 50% H_2_SO_4_ solution) and heating the plate on 120°C for 5 minutes. The lipid spots were scraped, mixed with Optiphase ‘Hi phase’ scintillation liquid (PerkinElmer-Wallac, Turku, Finland) in scintillation vials and the radioactivity was measured using a liquid scintillation counter, 1216 Rackbeta (PerkinElmer-Wallac, Turku, Finland). For the lipid mass quantification experiments, the band intensities of scanned HPTLC silica plates were analyzed and quantified using ImageJ software [35]. After lipid extraction, the cellular proteins were extracted with 0.1 M NaOH and the protein content was analyzed with the Lowry method [34]. Total counts per minute (cpm) obtained for the various experiments were normalized to total cellular protein. The results are displayed as the relative ^3^H-sphinganine or ^3^H-palmitic acid incorporation into various lipid species, or total lipid mass normalized to control cells.

### Reverse Transcription of RNA and Quantitative Real time PCR Analysis of GLTP and GlcCerS, GalCerS and LacCerS Expression in Treated Cells

HSF cells were treated identically to the experiments described above, but without ^3^H-sphinganine/^3^H-palmitic acid labeling. Instead of lipid extraction, total RNA was isolated directly from the cell dishes using a NucleoSpin RNA II (Macherey-Nagel, Germany) RNA-extraction kit, according to the manufacturer’s instruction. cDNA was obtained through reverse transcription of the purified RNA, which was carried out using M-MLV Reverse Transcriptase (Promega, Madison, WI, USA). The cDNA was amplified and quantified by performing quantitative real time PCR (qPCR). qPCR was performed by the staff at the Turku Centre for Biotechnology using the Applied Biosystems 7900 HT Fast Sequence Detection System, using the following primer pairs obtained from DNA Technology A/S (Risskov, Denmark): GLTP (sense) 5′-GAAGTACCATGGCTGGATCG-3′, GLTP (antisense) 5′-CAGACTTATAGGGTGCTGCGTA-3′, 18S rRNA (sense) 5′-GCAATTATTCCCCATGAACG-3′, 18S rRNA (antisense) 5′-GGGACTTAATCAACGCAAGC-3′, GlcCerS (sense) GTTTCAATCCAGAATGATCAGGT, GlcCerS (anti sense) AAGCATTCTGAAATTGGCTCA. GalCerS (sense) CTATGAAGCACTAGTGAAGGTTATCAA, GalCerS (anti sense) CCTTGTGAATTTCCGAAAGC, LacCerS (sense) TCATTGGAGGCCAAAAGACT, LacCerS (anti sense) TTCATGGCGATTACGGAAAG. The Universal ProbeLibrary probes #27 (for GLTP), #48 (for 18S rRNA) and #4 (for GlcCerS), #33 (for GalCerS) and #87 (for LacCerS) were obtained from Roche Diagnostics (Basel, Switzerland).

The mRNA expression levels of GLTP, GlcCerS, GalCerS and LacCerS were corrected to an 18S rRNA internal control. Results are shown as the relative mRNA expression normalized to control cells.

### Heat Shock and Induction of ER-stress

HSF cells were cultured in cell culture flasks until near confluency. The cell culture flasks, with tightened caps, were submerged in a 42°C water bath for 1 hour. Cells were then moved back into 37°C (5% CO_2_) and caps were loosened for a recovery period of 0−24 hours. ER-stress was induced by treating HSF cells with tunicamycin (10 µg/ml) for 24 hours. All cells were harvested simultaneously, and analyzed as described above.

### Statistical Analysis

The statistical two-tailed Student t-tests were applied to the data and is indicated with one asterisk (*), p<0.05 two asterisks (**), p<0.01 and three asterisks (***), p<0.005.

## Results

### Human GLTP Levels Increase in Conjunction with Increased GlcCer Levels

The fungal toxin BFA inhibits vesicular transfer of proteins and lipids to the plasma membrane by inducing retrograde protein transport from the Golgi apparatus to the ER. This effect in turn results in the fusion of these organelles, creating a Golgi-ER complex and leaving the trans-Golgi network fused with late endosomes [36]. Previously, it has been demonstrated that BFA treatment increases the incorporation of radiolabeled precursors into GlcCer, GalCer, LacCer and the gangliosides GM3 and GD3 [30], [31]. Monensin is a monovalent cationophore, which is also known to interfere with vesicular transport through the Golgi apparatus, affecting distal Golgi function because of swelling of its cisternae [37]. Monensin has been shown to inhibit the synthesis of sphingomyelin (SM) while increasing levels of radiolabeled precursor incorporation into GlcCer, GalCer and ceramide in cells [38].

**Figure 1:**
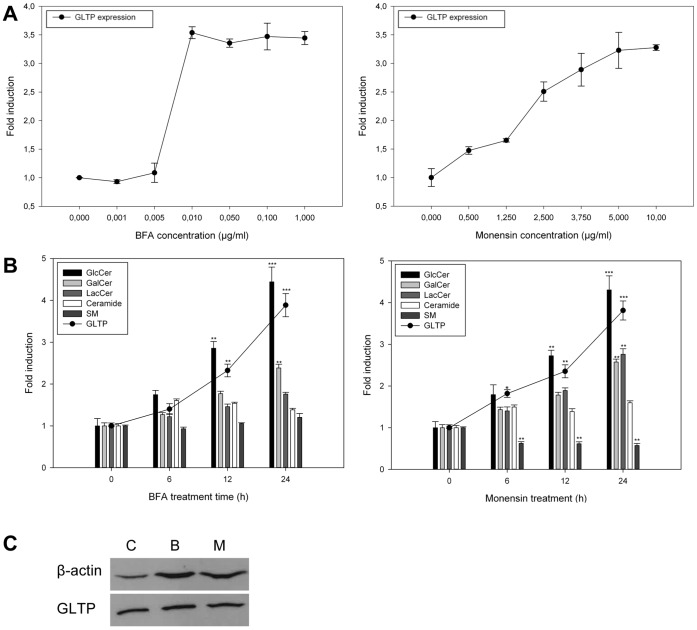
GLTP expression, GlcCer, Galcer, LacCer, ceramide and sphingomyelin synthesis in HSF cells as a function of BFA or monensin treatment. **A)** HFS cells were treated with BFA (left panel) or monensin (right panel) with increasing concentrations for 24 hours. The GLTP mRNA expression levels were analyzed using qPCR and corrected to an 18S rRNA internal control. **B)** qPCR analysis of GLTP expression (filled circles) and sphingolipid levels in HSF cells treated with BFA (0.01 µg/ml, left panel) or monensin (5 µg/ml, right panel) for 6, 12 and 24 hours, ^3^H-sphinganine incorporation into the sphingolipids was analyzed using TLC. qPCR results are expressed as means +/− SD of at least three independent experiments. The data for the incorporation of the radiolabeled ^3^H-sphinganine are from at least three different experiments, and the results are normalized to the controls. Two asterisks (**), p<0.01 and three asterisks (***), p<0.005 indicate the statistical significance compared to the controls. **C)** Western blot analysis of GLTP levels in HSF cells treated with BFA (0.01 µg/ml) or monensin (5 µg/ml) for 24 hours. C = untreated control, B = BFA treatment, M = monensin treatment. β-Actin was used as a loading control. The representative blot shown here was chosen from one of three independent experiments with similar results.

In order to analyze how GLTP is affected by changes in GSL metabolism, we first examined if and how GLTP expression is affected in HSF cells as a result of treatment with BFA and monensin. We examined the expression of GLTP as a function of different concentrations of BFA and monensin after a 24-hour treatment (Figure 1A). GLTP expression levels were analyzed using reverse transcription qPCR, using 18S rRNA as an internal control. The results show that both compounds increase GLTP expression significantly in a concentration dependent manner. For BFA, GLTP expression reaches a plateau at concentrations as low as 0.01 µg/ml, whereas monensin induced GLTP expression appears to have a more linear increase, reaching a plateau at around 5 µg/ml.

Next, we compared the incorporation of ^3^H-sphinganine into GlcCer, GalCer, LacCer, SM and ceramide and the GLTP expression, as a function of BFA and monensin treatment time (Figure 1B). This was done at concentrations of 0.01 µg/ml for BFA (Figure 1B, right panel) and monensin at 5 µg/ml (Figure 1B, left panel), in accordance with the results from the previous experiment. Both BFA and monensin induce a clear and significant increase in GLTP expression that is time dependent (Figure 1B, filled circles).

As seen in Figure 1B, treatment of HSF cells with BFA (left panel) results in an over four-fold increased incorporation of ^3^H-sphinganine into GlcCer. The synthesis of GalCer after 24 h of BFA treatment was significantly higher than the control, however the increase was not at high as for GlcCer.

Treatment of HSF cells with monensin (right panel) also resulted in an over four-fold increased incorporation of ^3^H-sphinganine into GlcCer, smaller but similar increase for GalCer and LacCer after 24h. The synthesis of ceramide appeared to be somewhat increased in both monensin and BFA treated cells. In monensin treated cells, incorporation of ^3^H-sphinganine into SM is significantly reduced. It is believed that this is due to monensin inhibiting transport of ceramide to the site of SM synthesis in the Golgi (Figure 1B, left panel) [38]. Furthermore, this increase in GLTP expression correlates well with the GlcCer synthesis as seen by an increased incorporation of the ^3^H-sphinganine label into GlcCer. Western blot analysis of cells treated with BFA and monensin for 24 hours shows that the GLTP protein level is also increased (Figure 1C).

### BFA or Monensin Treatment Increases the Masses of Simple GSLs

**Figure 2:**
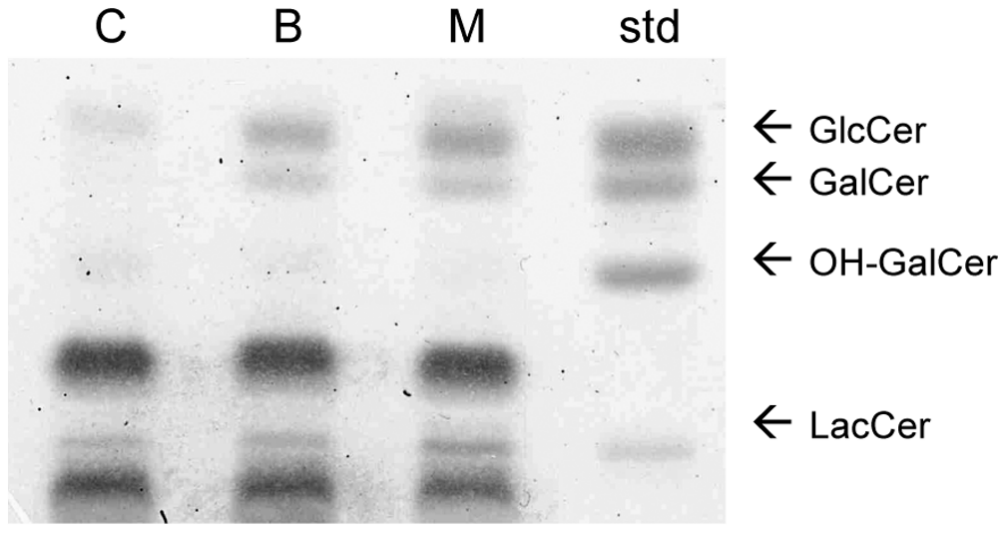
Changes in the mass of GlcCer, GalCer and LacCer. HSF cells were treated with BFA or monensin for 24 h and visualized with orcinol-sulphuric acid on a high performance TLC silica plate. OH-GalCer, hydroxylated GalCer. The representative TLC plate shown here was chosen from one of three independent experiments with similar results.

Radiolabeled precursor incorporation does not necessarily correspond with increases in total lipid mass. Throughout this study we therefore examined how the different treatments affected GSL masses with a traditional TLC approach. We show that both BFA or monesin treatment for 24 h results in an increase of total GlcCer, GalCer and LacCer masses (Figure 2). A representative high performance TLC plate (HPTLC), stained with the sugar sensitive orcinol-sulphuric acid spray, shows that the masses of GlcCer, GalCer, and LacCer indeed also change with the BFA and monensin treatments.

### The Expression of Glycosphingolipid Synthase Genes is Affected by BFA or Monensin Treatment

**Figure 3:**
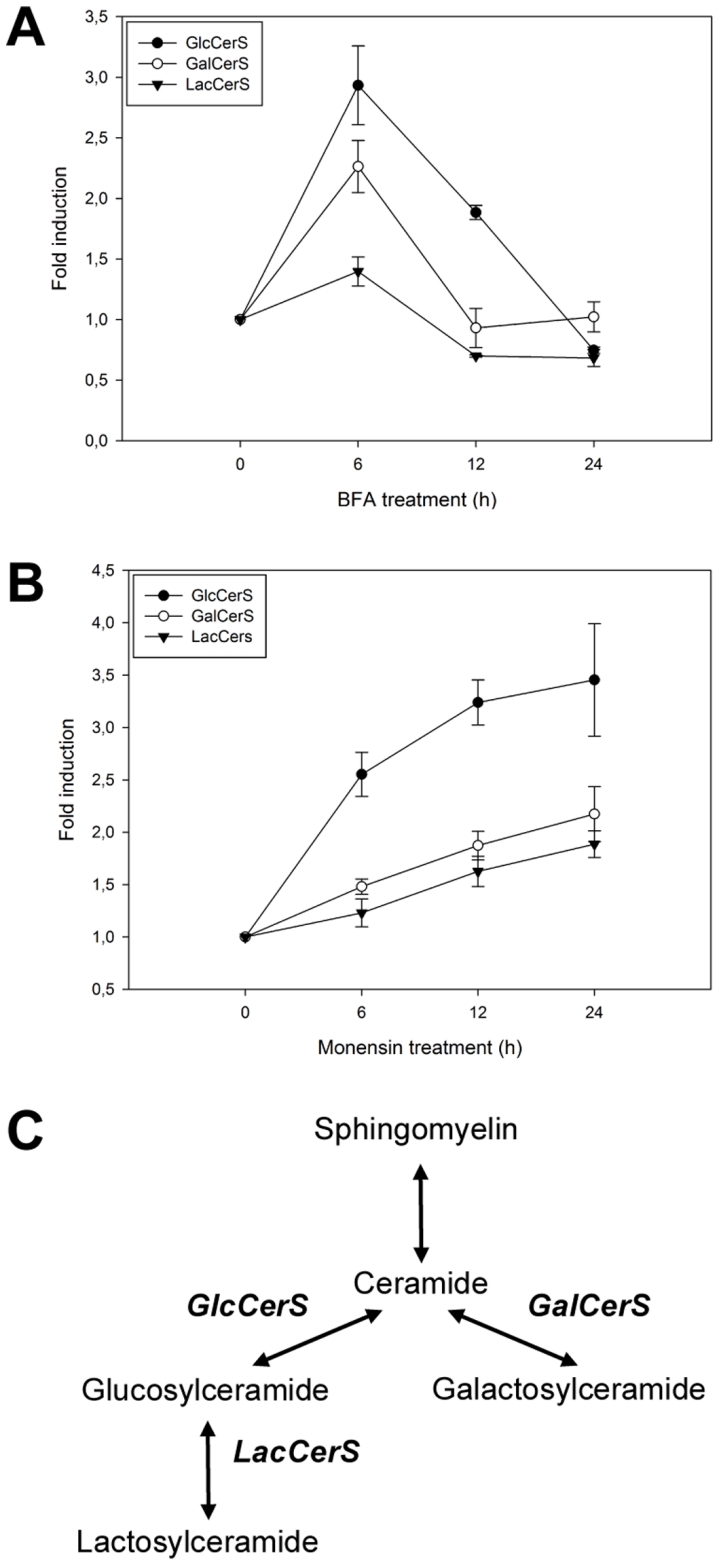
Effects of BFA and monensin on GlcCerS, GalCerS and LacCerS mRNA expression levels. qPCR analysis of the expression levels in cells treated with **A)** BFA (0.01 µg/ml) and **B)** monensin (5 µg/ml) for 0, 6, 12 and 24 hours. The qPCR results are expressed as means +/− SD of three independent experiments. **C)** Scheme of the enzymes in the sphingolipid metabolism analyzed in this study, *GalCerS* (galactosylceramide synthase) *GlcCerS* (glucosylceramide synthase) and *LacCerS* (lactosylceramide synthase).

The expression of GlcCerS, GalCerS and LacCerS in the BFA or monensin treated HSF cells were also analyzed using qPCR (Figure 3A and 3B). Interestingly, for BFA treated cells, the gene expressions were highest after 6 hours of treatment; subsequently the mRNA levels started to diminish and reached the starting level after 24 hours (Figure 3A). For the monensin treated HSF cells on the other hand, the GlcCerS expression raised and appears to flatten out after 24 hours of treatment. GalCerS and LacCerS followed the same increasing trend, but only with a 2-fold overall increase after 24 h of treatment (Figure 3B). A scheme of the enzymes in the sphingolipid metabolism analyzed in this study is shown in Figure 3C.

### Increased Accumulation of GlcCer in the Lysosomes Does not Affect GLTP Expression Levels

The data presented here and in previous work show that treatment of cells with monensin or BFA lead to the increased synthesis and/or accumulation of newly synthesized GlcCer in the Golgi and the ER, or in the case of cells treated with BFA, in the fused ER-Golgi complex [39]. In BFA treated cells, the accumulation is localized to these organelles, due to the inhibition of vesicular transport towards the cell surface and other cellular compartments. It is unclear whether monensin gives rise to a similar accumulation of GSLs, but our results show that GlcCer synthesis is significantly increased, and GalCer and LacCer to a lesser extent. This leads us to believe that increased amounts of GlcCer in the Golgi and/or the ER in these cells could be sensed by GLTP.

**Figure 4:**
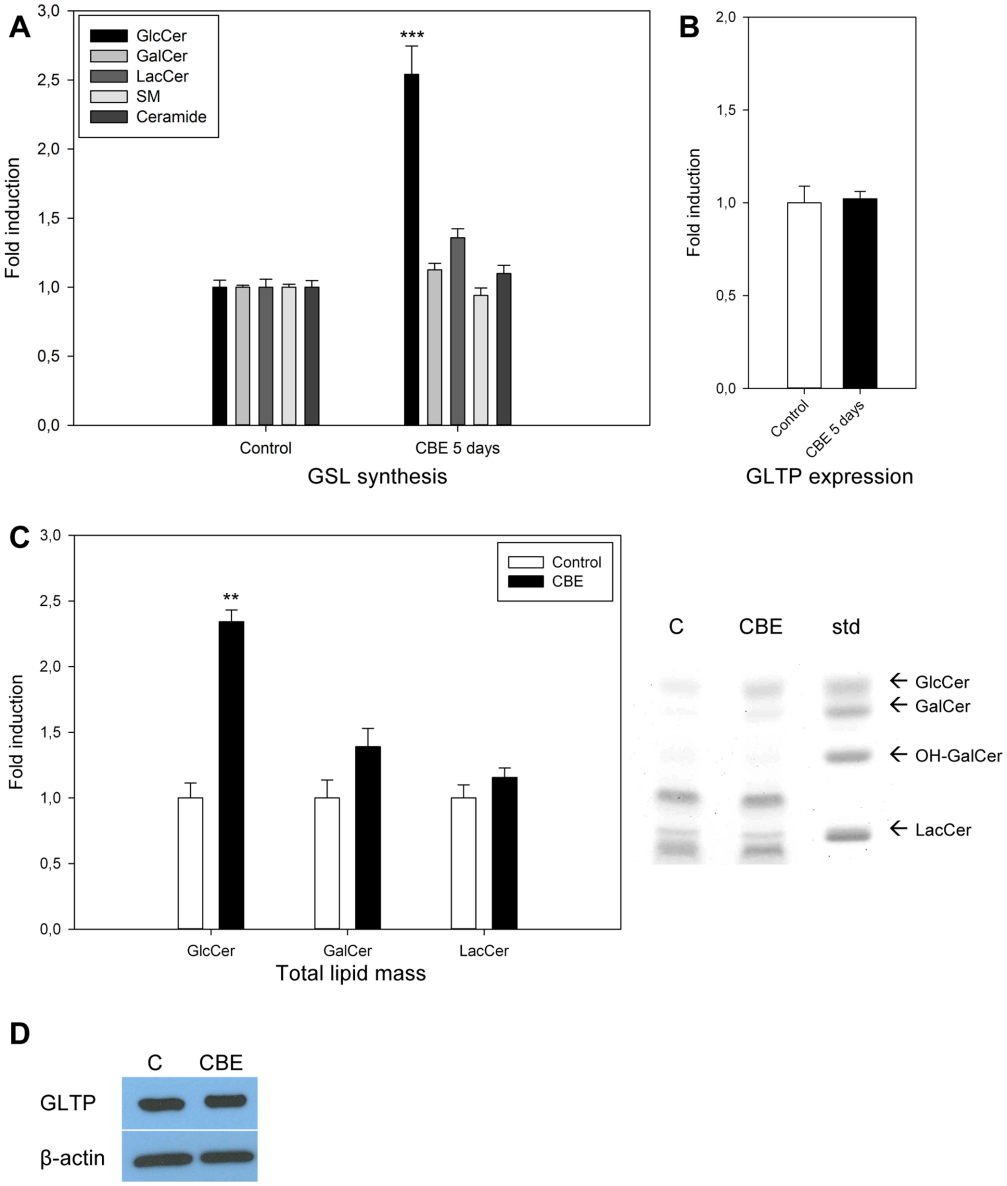
Effect of CBE treatment on GLTP mRNA and protein levels. HSF cells were treated with CBE (250 µM) for 5 days. **A)** Simple sphingolipid levels were determined by ^3^H-sphinganine incorporation and TLC analysis. Incorporation of ^3^H-sphinganine into GlcCer, GalCer, LacCer, SM and ceramide in untreated controls compared to CBE treated cells. GlcCer (***) levels in CBE treated cells are significantly higher than their controls (p<0.005). **B)** The GLTP mRNA expression was determined by qPCR in CBE treated HSF cells. Results are expressed as means +/− SD of three independent qPCR experiments. **C)** The total lipid mass of GlcCer, GalCer and LacCer as visualized by orcinol-sulphuric acid on a HPTLC plate, as well as lipid band intensities semi-quantified using ImageJ software and normalized to the controls. GlcCer (**) levels in CBE treated cells are significantly higher than their controls (p<0.01). **D)** Western blot of cells treated as described above, C = untreated controls, CBE = 5 day CBE treatment (250 µM). β-Actin was used as a loading control.

To examine whether GlcCer accumulation in the lysosomes, caused by inhibited GlcCer degradation will affect the GLTP expression, HSF cells were treated with conduritol-B-epoxide (CBE). CBE is an inhibitor of beta-glucosidase, an enzyme that degrades GlcCer in the lysosomes, consequently mimicking Gaucher disease [32]. A significant incorporation of ^3^H-sphinganine into GlcCer is shown in Figure 4A as well as an increase in the total GlcCer mass, Figure 4C. However, CBE treatment does neither affect the GLTP mRNA nor the protein levels (Figure 4B and 4D). This suggests that the GLTP expression correlates with GlcCer in the ER/Golgi compartments, rather than with the accumulated levels of GlcCer in the lysosomes. Responses in GLTP in cells with different GSL degradation disorders are currently being investigated.

### Co-treatment of BFA and Monensin Treated Cells with Inhibitors of GlcCer Synthesis Normalize GLTP Expression

**Figure 5:**
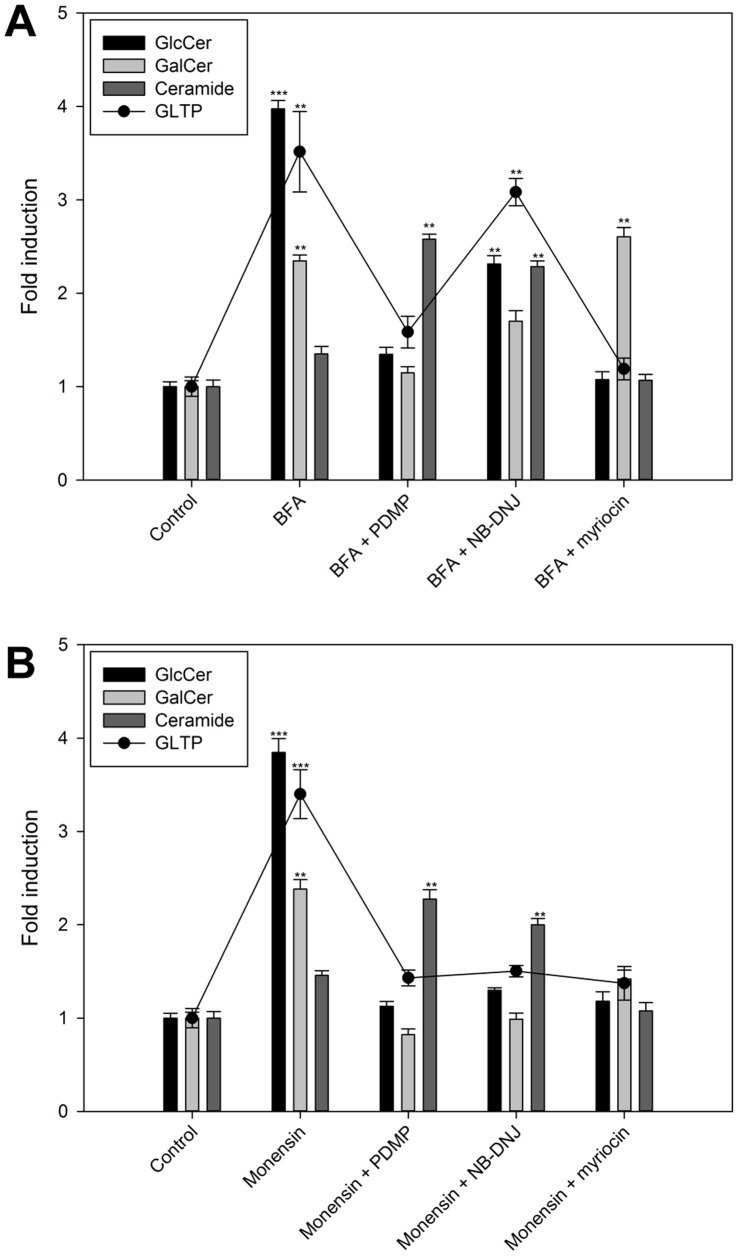
GlcCer, GalCer and Cer and GLTP mRNA levels in HSF cells co-treated with BFA/monensin and different GlcCer synthesis inhibitors. **A)** ^3^H-sphinganine incorporation and GLTP mRNA levels (filled circles) in HSF cells treated with either BFA (0.01 µg/ml) as well as HSF cells co-treated with BFA in addition to PDMP (50 µM), NB-DNJ (250 µM) and myriocin (25 µM). **B)** ^3^H-sphinganine incorporation and GLTP mRNA levels (filled circles) in HSF cells treated with either monensin (5 µg/ml) as well as HSF cells co-treated with monensin in addition to PDMP (50 µM), NB-DNJ (250 µM) and myriocin (25 µM). HSF cells treated with myriocin were labeled with ^3^H-palmitic acid. The results are expressed as means +/− SD of three independent experiments. Two asterisks (**), p<0.01 and three asterisks (***), p<0.005 indicate the statistical significance compared to the controls.

The results above suggest that an increase in the synthesis of GlcCer appears to be sensed by GLTP. To examine whether GLTP is linked to GlcCer levels, cells were co-treated with two GlcCer synthesis inhibitors and an inhibitor of serine palmitoyltransferase (Figure 5). If BFA and monensin treatment cause GlcCer accumulation and an increase in GLTP expression, then a simultaneous blocking of GlcCer synthesis with NB-DNJ an PDMP should result in less GlcCer accumulation, and consequently lower GLTP expression.

With the exception of BFA/NB-DNJ treated cells, all co-treatment experiments, as expected, lowered GlcCer levels close to that of the control with no BFA treatment (Figure 5A, black bars). In BFA/NB-DNJ treated cells, there was only a partial reduction in GlcCer levels compared to the high BFA control (Figure 5A). The GalCer synthesis was also reduced to the normal levels in BFA/PDMP treated cells, but only again partially reduced for the BFA/NB-DNJ treated cells (Figure 5A light grey bars). The response in the ceramide levels was inversely correlated with the GlcCer and GalCer response, so that the levels of ceramide went up when the synthesis of GlcCer was blocked with both PDMP and NB-DNJ (Figure 5A, dark grey bars).

When monensin was used to block the intracellular membrane trafficking mechanisms, the response in the GlcCer, GalCer and ceramide levels were similar to the BFA treatments. However, for the monensin+NB-DNJ or PDMP treatment the level of GlcCer and GalCer also came down to the normal levels (Figure 5B). Ceramide levels are comparable to the BFA experiment, where ceramide levels increased when the synthesis of GlcCer and GalCer was inhibited.

In the serine palmitoyltransferase inhibitor myriocin experiments (Figure 5A & 5B), cells were labeled using ^3^H-palmitic acid instead of ^3^H-sphinganine, because myriocin inhibits the reaction between palmitoyl-CoA and L-serine, one step before the ceramide synthase catalyzed reaction between sphinganine and fatty-acyl CoA. The GlcCer and ceramide amounts for the BFA/myriocin treated HSF cells were at the control levels, whereas the GalCer levels were not affected by the myriocin inhibitor and remained as high as for the BFA only treated cells (Figure 5A). Monensin and myriocin treated cells had however all lipid levels comparable to that of the controls, (Figure 5B). Furthermore, the GLTP mRNA expression correlates (Figure 5, filled circles) with the GlcCer and GalCer synthesis, but not with the ceramide synthesis. The statistical significance compared to the respective controls is indicated with asterisks.

### Effects of GSL Biosynthesis Inhibitors on GlcCer, GalCer, GLTP Protein and mRNA Levels

**Figure 6:**
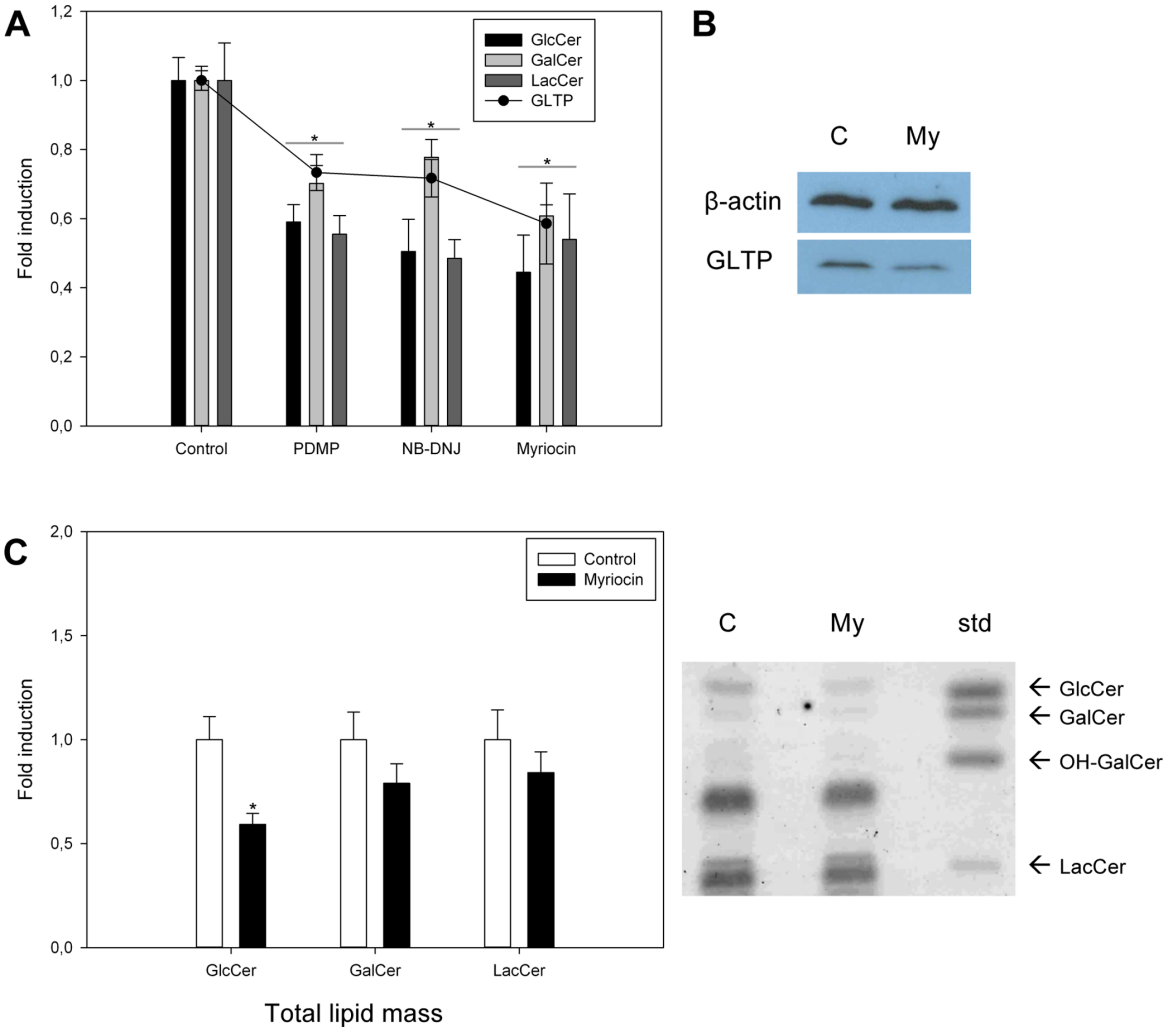
Effect of GSL synthesis inhibitors on GlcCer, Galcer, LacCer and GLTP protein and mRNA levels. **A)** ^3^H-palmitic acid incorporation into GlcCer, GalCer and LacCer and GLTP expression levels (filled circles) in HSF cells treated with PDMP (50 µM), NB-DNJ (250 µM) or myriocin (25 µM) for 24 hours. qPCR and precursor incorporation results are expressed as means +/− SD of at least three independent experiments. The asterisk (*), p<0.01 indicate the statistical significance compared to the controls. **B)** GLTP levels analyzed by Western blot in HSF cells treated with myriocin (25 µM) for 72 hours. β-actin was used as a loading control. C = control and My = myriocin. **C)** The total lipid mass of GlcCer, GalCer and LacCer as visualized by orcinol-sulphuric acid spray on a high performance TLC plate, in HSF cells treated with myriocin (25 µM) for 72 hours. The lipid band intensities on the plate were also semi-quantified using ImageJ software and normalized to the intensities of the control spots. OH-GalCer, hydroxylated GalCer.

When HSF cells were treated with the GSL synthesis inhibitors alone, all inhibitors caused the expected reduced GlcCer and GalCer and GLTP expression levels (Figure 6A). Western blot analysis (Figure 6B) and total lipid mass quantification (Figure 6C) show that both the GlcCer mass and GLTP levels are significantly reduced when cells are treated with myriocin for 72 hours. GalCer and LacCer were not reduced to the same extent as GlcCer.

### GlcCer Synthase Down-regulation

**Figure 7:**
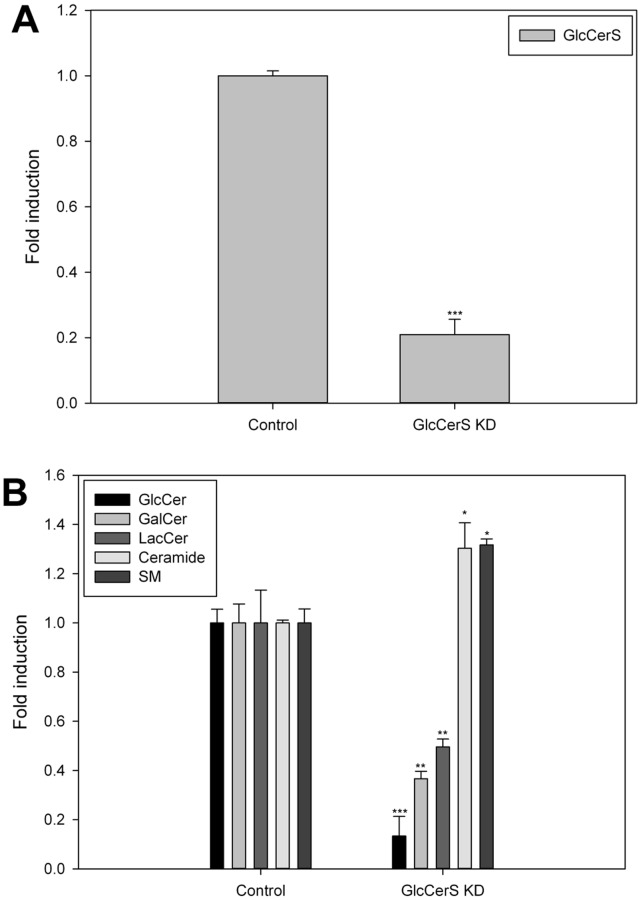
Knockdown of GlcCerS and effects on GSLs in HSF cells. **A)** qPCR assessment of the GlcCerS levels induced by silencing the GlcCerS gene normalized to the levels in mock-transfected HSF cells. **B)** GlcCer, GalCer, LacCer, Cer and SM levels were measured in HSF cells labeled with ^3^H-sphinganine for XX hours, and normalized to the levels in normal control HSF cells. The significance in the changes of the lipid levels is indicated with asterisks. One asterisk (*), p<0.05, two asterisks (**), p<0.01 and three asterisks (***), p<0.005 indicate the statistical significance compared to the controls.

**Figure 8:**
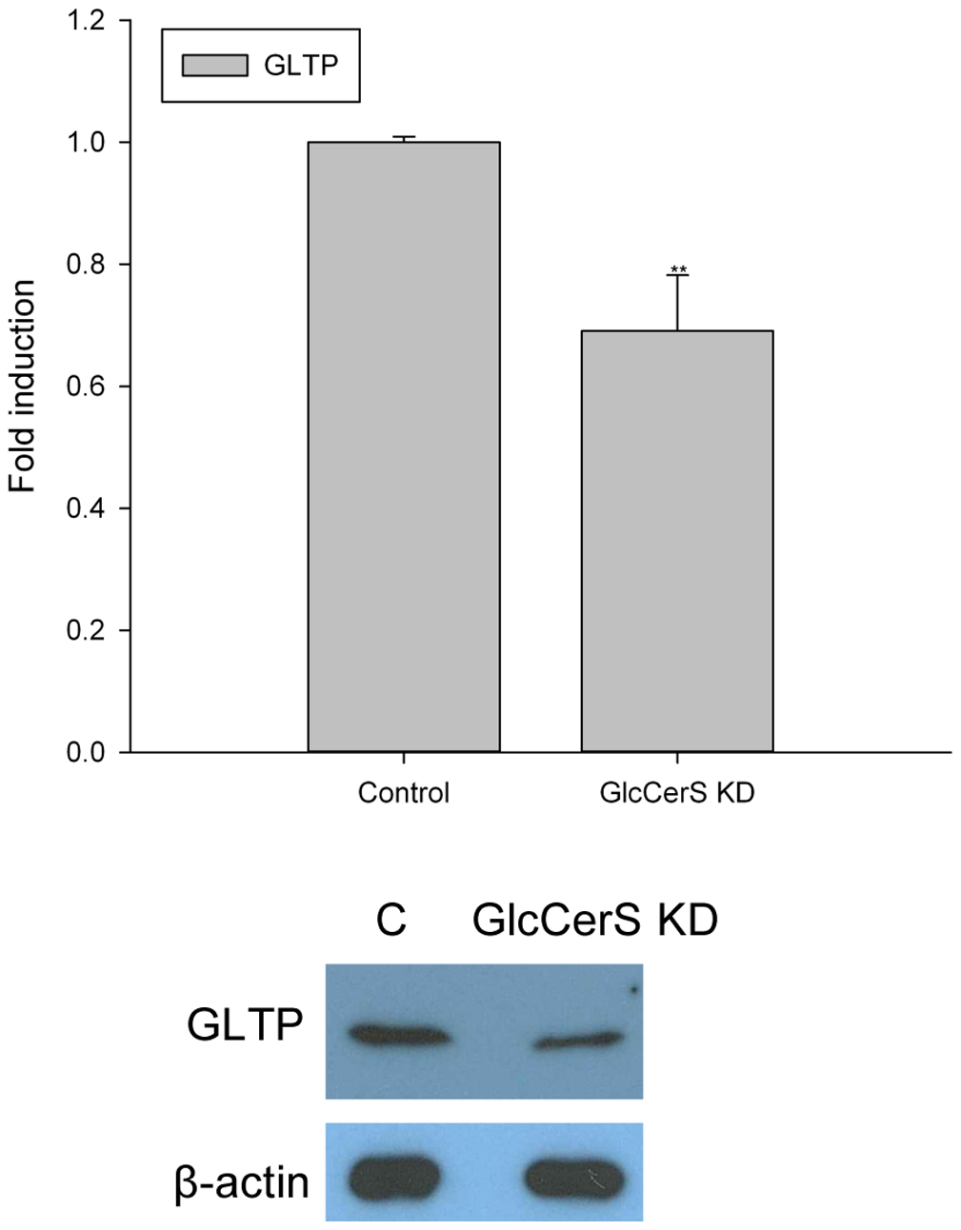
Expression of GLTP in GlcCerS knockdown HSF cells. The GLTP levels were analyzed with both qPCR and Western blot in HSF cells with 80% down-regulated expression of the GlcCerS gene. The GLTP gene level was normalized to the level in mock-transfected HSF cells, and β-actin was used as a loading control in the GLTP protein expression analysis.

GlcCer is synthesized from ceramide by GlcCerS at the cytosolic leaflet of early Golgi membranes. GlcCerS (also called UGCG, NM_003358) was down-regulated using the manufacturers instructions. The expression of the GlcCerS gene was down-regulated by approximately 80% compared to the expression of GlcCerS in normal HSF cells (Fig. 7A). The GlcCerS expression levels were analyzed using reverse transcription qPCR. The synthesis of GlcCer, GalCer and LacCer was significantly decreased, and the Cer and SM levels increased (Fig. 7B). Inline with the GlcCer synthesis inhibitor experiments (Figure 6A), the expression of GLTP was significantly reduced by the down-regulation of GlcCerS, both on mRNA and protein levels (Figure 8).

### Effects on GLTP in GlcCerS KD Cells with Impaired Intracellular Membrane Trafficking (BFA & Monensin)

**Figure 9:**
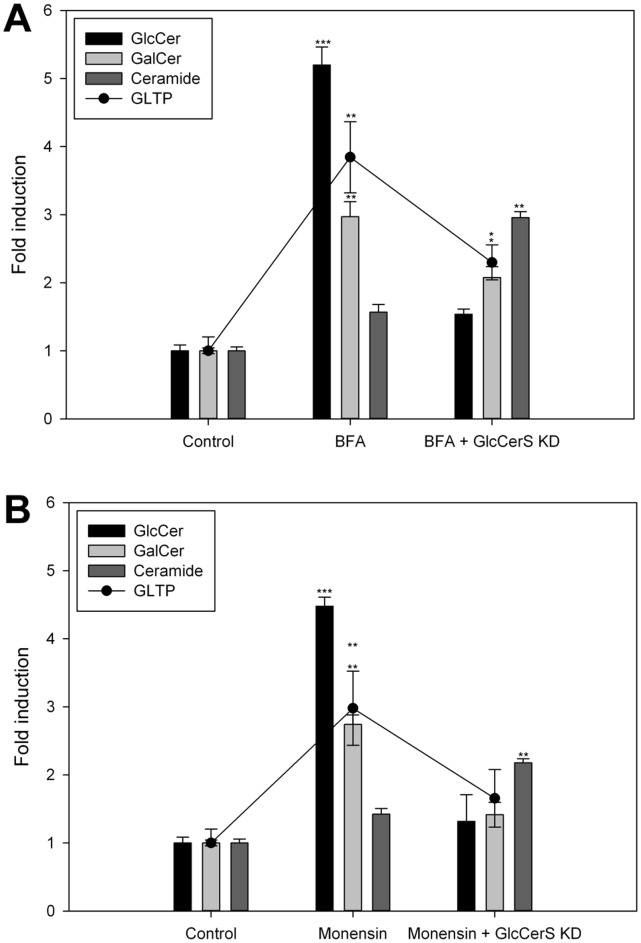
Expression of GLTP in GlcCerS knockdown cells with impaired intracellular membrane trafficking caused by BFA and monensin. **A)** ^3^H-sphinganine incorporation into GlcCer, GalCer and Cer as well as GLTP mRNA levels (filled circles) in GlcCerS KD HSF cells treated with BFA (0.01 µg/ml) and **B)** treated with monensin (5 µg/ml). Two asterisks (**), p<0.01 and three asterisks (***), p<0.005 indicate the statistical significance compared to the controls.

We also analyzed the effects that the knockdown of GlcCerS had on the expression levels of GLTP in BFA and monensin treated cells, analogous with the GlcCer synthesis inhibitor experiments (compare to Figures 5A & 5B). When less GlcCer was synthesized (80% KD) and the vesicular transport blocked by BFA, the response in GLTP was also lowered (Figure 9A). This was also observed in the monesin treated GlcCerS KD cells (Figure 9B). Also here the ceramide levels increased when the synthesis of GlcCer down-regulated, an observation similar to the previous inhibitor experiments. The statistical significance compared to the respective controls is indicated with asterisks.

### ER and Heat Shock Stress Controls do not Affect GLTP Levels

**Figure 10:**
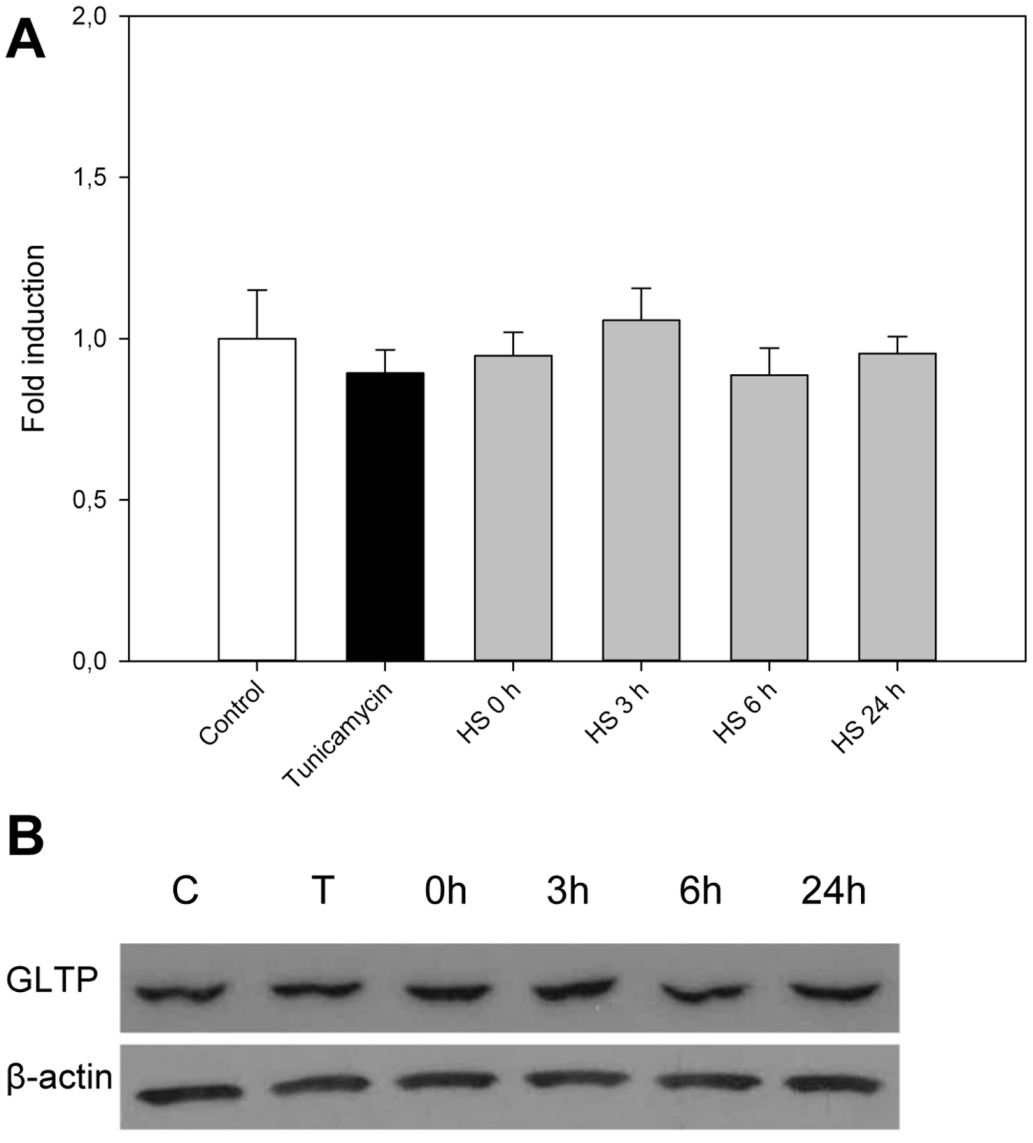
GLTP response to tunicamycin and heat shock. **A)** GLTP mRNA expression determined by qPCR in cells undergoing heat shock and ER-stress. HSF cells were either heat shocked at 42°C for one hour, following different recovery periods (0–24 hours) at 37°C, or treated with tunicamycin (10 µg/ml) for 24 hours. Results are expressed as means +/− SD of three independent experiments. **B)** GLTP protein levels in HSF cells analyzed with Western blot after one hour heat shock followed by recovery, or tunicamycin at 10 µg/ml for 24 hours. C = control and T = tunicamycin, β-actin was used as a loading control.

Since both monensin and BFA cause structural changes in the ER and Golgi compartments and subsequently place the cell under great stress, we examined how two other types of stresses affect GLTP. HSF cells were either heat shocked or treated with tunicamycin, a nucleoside antibiotic that inhibits protein glycosylation and induces ER-stress [40]. In Figure 10 it can be seen that neither heat shocking nor tunicamycin cause any significant changes in GLTP expression levels. This further strengthens the argument that the accumulation of simple glycosphingolipids indeed could be the cause of the increased GLTP amounts and not the ER-stress *per se*.

## Discussion

Eukaryotes that do not encode the glucosylceramide synthase neither produce GLTPs, however cells that can make glucosylceramide also express GLTPs [20], [41]. This fundamental genetic relationship has prompted us to investigate if the synthesis of GlcCer can affect the expression of GLTP in mammalian cells. We have previously determined that overexpression of GLTP in HeLa cells, and in this work HSF cells (data not shown) show a significant increase in the synthesis of GlcCer and a decrease in the SM synthesis, however no changes were detected in the sphingolipid synthesis in GLTP-knockdown cells compared to control cells [14]. No increase in the GalCer levels was observed in either cell types. Only ceramide has been shown to induce GLTP promoter activity and raised transcription levels *in vivo* [42]. Other sphingolipid metabolites, such as GlcCer, sulfatide, gangliosides GM1, sphingosine and sphingoline-1-phosphate did not trigger any changes in the GLTP transcription. Here we wanted to study if GLTP levels would be affected by an GlcCer accumulation.

*In vitro* studies clearly suggest the capability of GLTP to accelerate a non-energy required glycolipid transport between membranes. Cellular distribution studies done in HeLa cells overexpressing GLTP show that GLTP localizes to the cytosol [14]. This localization is of importance when considering which glycolipid species are likely to associate with GLTP in cells. The synthesis of glycolipids takes place in the ER and the Golgi apparatus. Most GSLs are synthesized on the luminal side of the Golgi membranes, with the exception of GlcCer that is synthesized on the cytosolic side of the Golgi [4]. The site of synthesis of complex GSLs would render them inaccessible to GLTP, whereas GLTP would have access to GlcCer. GlcCer transport to distal sites of higher GSL synthesis in the Golgi has been shown to be FAPP2 dependent [24], [43]. Halter et al. showed that GlcCer reaches the late Golgi through the ER where FAPP2 is responsible for mediating the transfer of GlcCer from the early Golgi back to the ER [43]. Therefore, it is possible that GLTP would not be involved in the transfer of GlcCer from the Golgi, because GLTP does not contain any known Golgi targeting domains and has not been shown to associate with the Golgi. Unlike GLTP, FAPP2 has a pleckstrin homology (PH) domain that targets it to phosphatidyl-inositol-4-phosphate (PI4P) in the late Golgi [23]. Instead, GLTP has been shown to contain a FFAT-like motif [26] that interacts with the ER membrane protein VAP-A *in vitro* [27], [44], [45]. FFAT-like motifs have been identified in several other ER-targeting cytosolic proteins, including the ceramide transfer protein, CERT, that transports ceramide from the ER to the Golgi and is required for SM synthesis [46]. It is therefore possible that GLTP might be targeted to the ER through its FFAT-like motif where it would interact with GlcCer, possibly functioning as a regulatory sensor for these glycolipids. GLTPs function in GlcCer trafficking is still not ruled out. One possible role for GLTP would be to catalyze the transfer of GlcCer from the Golgi and the ER to the plasma membrane. A function already proposed early on by both Sasaki and Warnock and co-workers [47], [48], and is based on the finding that the non-vesicular transport of GlcCer to the plasma membrane still continues after FAPP2 down regulation [24], [43].

Here, we show that BFA and monensin treatment of HSF cells results in higher GLTP mRNA expression and higher GLTP protein levels. Monensin and BFA both inhibit vesicular transport and lead to an increased synthesis of sphingolipids in the ER and the Golgi apparatus [49]. While the precise site of GSL accumulation in monensin treated cells is unclear, BFA treatment yields GSL accumulation in a fused ER/Golgi complex [36]. From our CBE treatment results, it is apparent that an accumulation of GlcCer in the lysosomes does not increase GLTP expression. Presumably, an increased synthesis or accumulation of GlcCer in the ER or Golgi is required for GLTP expression levels to increase.

Increase in GLTP expression as a result of BFA and monensin treatment are both time and concentration dependent. It is apparent that, in these treatments, there is a strong correlation between the precursor incorporation into GlcCer and the increase in GLTP mRNA over time. GlcCerS expression in monensin treated cells is rapidly increased and stays elevated throughout the treatment. In BFA treated cells, GlcCerS expression peaks at 6 hours, where after it starts to diminish and is ultimately reduced to normal values after 24 hours. However, regardless of the GlcCerS expression patterns observed, GlcCer labeling is still increased throughout the treatment.

To strengthen our assumption that the rise in GLTP levels, as a result of BFA and monensin treatments, was due to changes in GSL metabolism, we used specific inhibitors of GSL synthesis to dampen the BFA and monensin effects observed. To achieve similar lowering of GlcCer we also studied the effects on GLTP by down-regulating the GlcCerS gene expression by RNA interference.

BFA and monensin treated HSF cells were co-treated with the GlcCer synthesis inhibitors PDMP and NB-DNJ and the serine palmitoyltransferase inhibitor myriocin, allowing us to analyze if the accumulation of GlcCer could be inhibited, and subsequently we would be able to determine if this would also yield lower GLTP levels. While PDMP and NB-DNJ are inhibitors of GlcCer synthesis, myriocin affects the biosynthesis of all sphingolipids, including sphingomyelin, by inhibiting the first step in sphingosine biosynthesis [33]. Co-treatment with the inhibitors indeed resulted in a lowered GLTP expression and GlcCer labeling and to some extent also GalCer labeling. Knockdown of GlcCerS showed a similar pattern in both the lipid levels and in how GLTP is expressed.

GLTP expression was almost completely normalized in all of the inhibitor co-treatment experiments as well as for the GlcCer siRNA experiments. The exception was BFA/NB-DNJ co-treated cells. Here, GLTP expression remained comparable to that of cells treated with BFA alone, while GlcCer labeling was only partially reduced. It is possible that BFA partially inhibits NB-DNJ action, which would result in a diminished NB-DNJ activity.

It should be noted that PDMP interferes with BFA and negates some of its effects [50], [51]. PDMP blocks BFA-induced retrograde membrane transport from the Golgi to the ER, but does not interfere with the BFA-induced inhibition of the binding of ADP-ribosylation factor and the coatomer component beta-coat protein to Golgi membranes [50], [51]. Vesicular transport is still inhibited from the Golgi to the plasma membrane during these conditions. In monensin treated cells, all GSL synthesis inhibitors normalized both radiolabeling of GlcCer and the GLTP expression almost fully, further strengthening the correlation between newly synthesized GlcCer levels and GLTP expression.

Does GalCer levels also affect the GLTP expression? The GalCerS has been shown to be localized in the lumen of the ER [8]. However, it has been speculated that GalCer would be able to flip or be flipped by an active translocator to the cytosolic leaflet of the ER [52]. If this were the case, GalCer would also be rendered accessible to GLTP. Nevertheless, in BFA/myriocin treated cells, GalCer labeling remained high, while GLTP and GlcCer levels were normalized. It is therefore concluded that it is unlikely that GLTP reacts to GalCer levels in these experiments. We do not know why GalCer synthesis is not blocked in the BFA/myriocin treated cells. It could be possible since the synthesis of ceramide is not completely blocked by myriocin, that a fraction of ceramide is still available for the GalCerS in the ER compartment, but not for GlcCerS in the Golgi because of the blocked lipid trafficking caused by BFA. Another possibility could be that BFA interfere with myriocin and the blocking of the serine palmitoyltransferase is weakened. When treating cells using GSL biosynthesis inhibitors on their own, all inhibitors significantly reduced GlcCer and to a lesser extent GalCer and subsequently, GLTP levels below that of normal values. This further strengthens our conclusions that GLTP is capable of recognizing changes in the levels of GlcCer.

We also investigated whether heat shock and ER-stress affects GLTP expression. Hait et al. showed that sphingolipids are involved in thermal, chemical and other cell stress responses [51]. It is therefore likely that GLTP also could be affected by the cell stress response and that the stress the cells undergo during BFA and monensin treatment is in fact the reason for increased GLTP expression. Still, neither heat shock nor induction of ER-stress by tunicamycin treatment increases GLTP expression, suggesting that GLTP is not affected under these stress events in the cell. Clearly the role of GLTP under different stress events warrants more investigations.

In summary, our results show a strong correlation between GLTP expression levels and quantities of synthesized GlcCer. Addition of GlcCer, sulfatide, GM1, ceramide 1-phosphate, sphingosine 1-phosphate, dihydroceramide and sphingosine in the culture media of HeLa cells did not cause any changes in the GLTP transcription levels [42]. Ceramide was the only lipid added that triggered an increase in the GLTP promoter activity via Sp1/Sp3 transcription factors and raised transcript levels [42]. This further strengthens our conclusions that GLTP is sensitive to GSL precursor synthesis, and not GlcCer generated from GSLs from the degradation pathways. We cannot yet rule out that higher amounts of GalCer also would affect the expression of GLTP. It is tempting to speculate that perhaps GLTP with its ER targeting FFAT-like domain could direct GlcCer and GalCer away from ER to other destination, such as GlcCer to the plasma membrane, or to the Golgi for further glycosylation, and GalCer also to the Golgi to become sulfatide. Clearly further experiments are required to elucidate what role GLTP plays in the lipid sensing and transfer and whether GLTP is a player at the ER-Golgi interface, regulating the flow and branching of precursor glycosphingolipids. If GLTP plays such a role, it is likely that the action of GLTP is connected to the complex synthesis scheme of ceramide with the six different ceramide synthases [29].
