# Correction: Glycolipid Transfer Protein Expression Is Affected by Glycosphingolipid Synthesis

**DOI:** 10.1371/annotation/712bb339-6073-4e62-9f68-b285caedd913

**Published:** 2013-10-16

**Authors:** Matti A. Kjellberg, Peter Mattjus

The panels in Figure 1C were switched. Please see the corrected Figure 1 here: 

**Figure:**
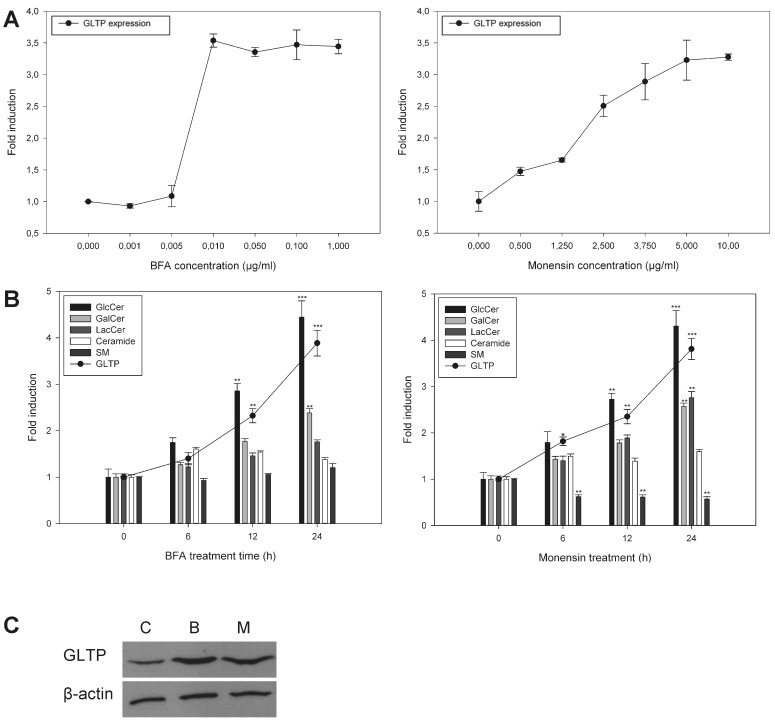


The correct Figure 7 caption reads:

A) qPCR assessment of the GlcCerS levels induced by silencing the GlcCerS gene normalized to the levels in mock-transfected HSF cells. B) GlcCer, GalCer, LacCer, Cer and SM levels were measured in HSF cells labeled with 3H-sphinganine for 24 hours, and normalized to the levels in normal control HSF cells. The significance in the changes of the lipid levels is indicated with asterisks. One asterisk (*), p<0.05, two asterisks (**), p<0.01 and three asterisks (***), p<0.005 indicate the statistical significance compared to the controls.

doi:10.1371/journal.pone.0070283.g007 

